# Intraoperative phenylephrine infusion to reduce perioperative shivering in lower segment caesarean section: A randomised controlled study

**DOI:** 10.1097/MD.0000000000033721

**Published:** 2023-05-12

**Authors:** Jessica Tan Sook Kuan, Qurratu Aini Musthafa, Farah Hanim Abdullah, Syarifah Noor Nazihah Sayed Masri

**Affiliations:** a Department of Anesthesiology and Intensive Care, Hospital Sultan Ismail, Johor Bharu, Malaysia; b Department of Anesthesiology and Intensive Care, Universiti Kebangsaan Malaysia Medical Centre, Bandar Tun Razak, Cheras, Kuala Lumpur, Malaysia.

**Keywords:** anesthesia, caesarean section, hypotension, hypothermia, phenylephrine, shivering, spinal

## Abstract

Shivering is described as an involuntary, repetitive activity of the skeletal muscles that can have deleterious effects on anaesthetized patients. This study aimed to evaluate the effectiveness of phenylephrine infusion in preventing perioperative shivering in patients undergoing lower segment cesarean section under spinal anesthesia and to observe the change in the patient’s core temperature between the study and control groups.

**Methods:**

A total of 118 patients scheduled for elective lower segment cesarean section under spinal anesthesia were recruited for this prospective, double-blind, randomized controlled study. The patients were randomized into 2 groups with 59 patients per group. The phenylephrine Group received phenylephrine infusion at a rate of 0.5 mcg/kg/minutes, while the Control Group received normal saline at an equivalent rate. Systolic and diastolic blood pressure, heart rate, core temperature, and the presence and intensity of shivering were recorded before induction and every 15 minutes intraoperatively and postoperatively.

**Results:**

The incidence of intraoperative shivering was significantly lower in the Phenylephrine Group compared to control group (29.1% vs 47.5% respectively; *P* = .044). Postoperatively, the Phenylephrine Group also had a lower incidence of shivering (34.5% vs 42.4%), but the difference was not statistically significant (*P* value = 0.391). There were no significant differences in the intensity of shivering between the 2 groups perioperatively, as well as in the systolic and diastolic blood pressure and core temperature. The phenylephrine Group showed a significantly lower heart rate at 15, 30, and 45 minutes after spinal block (*P* value = .005, .000, and .008, respectively), and at 0 and 30 minutes (*P* value = .004 and .020 respectively) in the recovery room. There were no significant differences in perioperative adverse events such as hypotension, hypertension, and bradycardia.

**Conclusion:**

Phenylephrine infusion reduces the incidence of perioperative shivering in lower segment cesarean sections under spinal anesthesia.

## 1. Introduction

Shivering is described as an involuntary, repetitive activity of skeletal muscles. It is activated when behavioral compensation and maximal vasoconstriction are insufficient to maintain core temperature.^[1]^ Studies have demonstrated that the incidence of shivering associated with central neuraxial block is at the range 40% to 70%.^[2,3]^ The incidence of shivering in obstetric patients with neuraxial blockade is between 40% to 60%.^[4,5]^

It is important for anesthesiologists to recognize and treat perioperative shivering and to prevent it, if possible, as shivering can have deleterious effects on anesthetized patients. Shivering can increase intraocular and intracranial pressure, oxygen consumption by 300% to 400%, carbon dioxide production, catecholamine release, metabolic heat production by up to 600%, metabolic rate by up to 400%, and lactic acidosis.^[1,6,7]^ These effects can be detrimental, especially in patients with a poor cardiopulmonary reserve. Shivering also causes patient discomfort and increases the likelihood of postoperative complications, such as infection, pain, and bleeding, leading to delayed wound healing and increased length of hospital stay.^[8,9]^

Perioperative hypothermia is defined as a core temperature of <33 to 35 °C, whereas the shivering threshold in non-anesthetized patients is 35.5 °C. Central neuraxial anesthesia impairs both central and peripheral thermoregulation as it increases the inter-threshold range by increasing the sweating threshold and decreasing the vasoconstriction threshold and shivering threshold (triggering core temperature) by approximately 0.5 to 0.6℃.^[1]^ Shivering during central neuraxial anesthesia is preceded by core hypothermia and vasoconstriction above the level of the block.^[10]^

Phenylephrine is an alpha-1 adrenergic receptor agonist with minimal to no beta-adrenergic activity. Therefore, it is mainly used intraoperatively to treat hypotension.^[11,12]^ Phenylephrine may also attenuate core hypothermia by inhibiting core to-peripheral redistribution of body heat during spinal anesthesia.^[13]^ The aim of this study was to evaluate the effectiveness of phenylephrine infusion in preventing perioperative shivering in patients undergoing lower segment cesarean section under spinal anesthesia and to observe the change in the patient’s core temperature between the study and control groups. The secondary objective of this study was to evaluate hemodynamic changes between the 2 groups. We hypothesized that phenylephrine infusion would reduce perioperative shivering in patients under spinal anesthesia.

## 2. Methods

Ethical approval for this study was provided by the Medical Research and Ethics Committee, Universiti Kebangsaan Malaysia Medical Centre, on 30^th^ May 2019 study approval number FF-2019-273). This study was registered at ClinicalTrials.gov (ID NCT04133961). This prospective, double-blind, parallel-group, randomized controlled study was conducted in the maternity operating theater of the Universiti Kebangsaan Malaysia Medical Centre from November 2019 to May 2020 in accordance with the Declaration of Helsinki 2013. All the patients who were screened and met the eligibility criteria were invited to participate in the trial, and all the enrolled patients provided written informed consent. Consent was requested from patients upon arrival to the operating suite for surgery or in the ward if they were admitted the night before surgery.

In total, 118 patients were recruited for this study. Inclusion criteria included patients aged 18 to 40 years old, American Society of Anesthesiologists physical status score II, and singleton pregnancy who were scheduled to undergo elective lower segment cesarean section under spinal anesthesia. Exclusion criteria included contraindications to phenylephrine, evidence of fever or infection, hypothermia (<35 °C) preoperatively, body mass index > 40 kg/m^2^, and height < 150 cm and patient’s refusal to participate in the study.

Using a computer-generated table, patients were randomly assigned to 1 of 2 groups, Phenylephrine (Group A) or Control (Group B) with a 1:1 allocation. Patient identifiers were attached to the opened envelopes and secured by a dedicated person, independent of the randomization procedure.

All patients fasted for at least 6 hours prior to surgery. They were given gastric acid aspiration prophylaxis prior to the surgery, which consisted of 150 mg oral ranitidine on the night prior to surgery and on the morning of surgery, and 30 mL of 0.3M sodium citrate oral solution on the morning of surgery. Patients and the attending anesthesiologist were blinded to the group to which the patients were allocated. Both phenylephrine (SAMARTH Frenin injection, India) and normal saline were prepared in 20 mL syringes by the investigator, and phenylephrine was diluted to a concentration of 100 mcg/mL. Patients in Phenylephrine Group (Group A) received phenylephrine infusion at rate of 0.5 mcg/kg/minute, which was equivalent to 0.3 mL/kg/hour, whereas Control group (Group B) patients received a saline infusion at a same rate of 0.3 mL/kg/hour. The syringes were labeled based on the same computer-generated randomized numbers, so that the attending anesthesiologist and patient did not know the content of the syringes.

Before induction of anesthesia, standard monitors, such as noninvasive systolic and diastolic blood pressure monitoring, continuous electrocardiography, and pulse oximetry, were applied to the patient. Baseline blood pressure, heart rate, oxygen saturation, and core body temperature were measured prior to induction. The ambient temperature of the operating theater was recorded, as measured by a standardized wall thermometer. The baseline core temperature of the patient was measured at the tympanic membrane using an infrared thermometer (Novo Temp TH50Z). Core temperature was measured every 15 minutes intraoperatively. All vital signs were recorded by an attending anesthesiologist who was blinded to the study.

A separate intravenous cannula was inserted to administer the study drugs. Co-loading with 500 mL of Hartmann’s solution was initiated in all patients prior to the commencement of spinal anesthesia. The patient was placed in a sitting position for spinal anesthesia. Spinal block was performed at the L3/L4 or L4/L5 levels using a 27-gauge spinal needle by the attending anesthesiologist under aseptic technique. Upon free flow of cerebrospinal fluid, 2 mL of 0.5% hyperbaric bupivacaine mixed with 0.1 mg morphine and fentanyl (15 µg) was administered to the spinal block. The time the spinal block was administered was recorded as “t_0_.” The patient was then positioned supine with left lateral tilt to prevent supine hypotension syndrome. Infusion of the study drug was initiated immediately after the block. The level of sensory blockade was tested using loss of sensation to cold, and anesthesia was considered adequate with a sensory block ≥ T4 dermatomal level. After the spinal block, the patient was covered with blankets in the upper body and lower limbs. All patients also received active warming via a forced-air warming device (Bair Hugger temperature set at 38 °C) placed in the upper part of the body. All fluids were prewarmed at 40 °C. Systolic and diastolic blood pressure and heart rate were recorded immediately after the spinal block and every 5 minutes for the first 30 minutes, followed by every 15 minutes thereafter. Other recorded intraoperative data included the total volume of fluids administered and estimated blood loss.

The primary outcome of the study was the incidence of shivering and changes in the patient’s core temperature, while the secondary outcomes were changes in hemodynamic parameters such as systolic and diastolic blood pressure and heart rate. All outcomes were assessed by the attending anesthesiologist both intraoperatively (from induction until completion of skin closure) and postoperatively in the recovery room.

The intensity of shivering was recorded by the attending anesthesiologist immediately after the spinal block and every 15 minutes during the surgery using the Bedside Shivering Assessment Scale.^[14]^ The Bedside Shivering Assessment Scale score ranges from 0 to 3, where 0 indicates no shivering, 1 indicates that shivering is localized to the neck and/or thorax, 2 indicates gross movement of the upper extremities, and 3 indicates gross movements of the trunk and upper and lower extremities. If the score was 2 or higher, intravenous pethidine 25 mg was administered after the baby had been delivered.

Adverse events, such as hypotension, hypertension, and bradycardia, were recorded and managed accordingly. Hypotension (defined as systolic blood pressure < 80% of the baseline) was treated with bolus doses of intravenous ephedrine (6 mg titrated to effect). Intravenous ephedrine were the first choice of rescue drugs to avoid bias. If the blood pressure remained low, an intravenous phenylephrine 100 µg bolus was administered, up to a maximum of 300 mcg. If hypertension (defined as systolic blood pressure > 120% of the baseline) occurred, the study drug infusion was terminated. If bradycardia (defined as a heart rate < 40 beats per minute) occurred, the study drug infusion was terminated, and intravenous atropine (0.5 mg) was administered if it was accompanied by hypotension. The dose and time of administration of all the rescue drugs were recorded. Patients were excluded from the study if they received more than 300 µg of phenylephrine bolus, conversion to general anesthesia, primary postpartum hemorrhage with blood loss more than 700 mL, surgical time exceeded 120 minutes, or if the drug infusion was terminated earlier during the surgery.

Drug infusion was terminated at the end of skin closure. Surgical time was defined as the time from the start of the surgical incision to completion of skin closure. The patient was transferred to the recovery room after surgery, and a warming blanket covering the entire body was applied. The ambient temperature of the recovery room was recorded. The patient’s core temperature, blood pressure, and heart rate were monitored every 15 minutes until the patient was discharged from the recovery room.

The sample size was estimated using the PS Power and Sample Size Calculations program by Dupont and Plummer (2009), based on the incidence of shivering among patients who received phenylephrine (Hilton et al^[15]^, 22.5%). An estimated sample size of 54 patients in each group was obtained, with the power of this study set at 80% and the α value set at 0.05. Considering a 10% dropout rate for this study, the estimated sample size was 59 patients in each group.

All data were analyzed using SPSS version 22.0 (IBM Corp, Armonk, NY). Results are presented as mean ± standard deviation, median with interquartile range, or frequency (percentages), where appropriate. An independent *t* test was used to analyze continuous data, whereas the chi-square test was used to analyze non-parametric data with *P* < .05 considered significant.

## 3. Results

**Figure 1.**
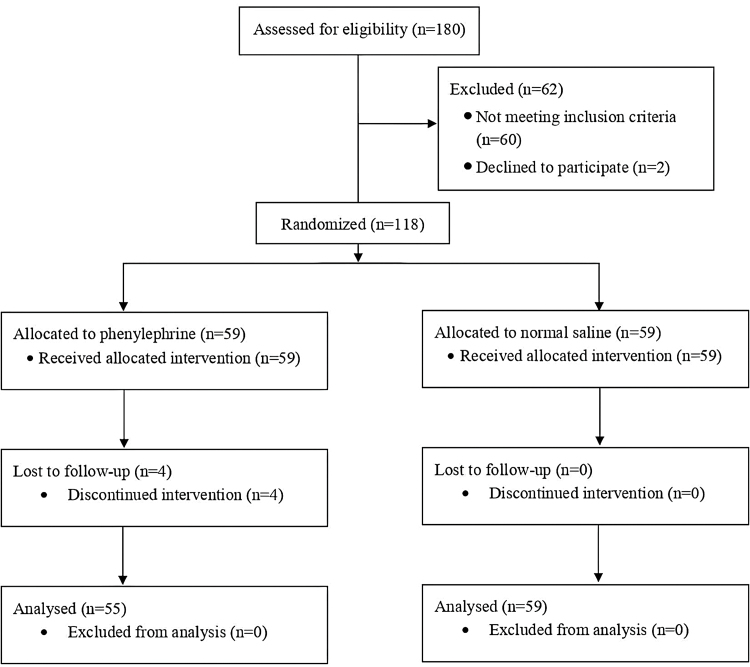
CONSORT diagram.

A total of 118 patients were recruited for this study, with 59 patients in each group. In total, 114 patients were included in the final analysis, with 55 and 59 patients in the phenylephrine and control groups, respectively. There were 4 drop-outs in the Phenylephrine Group, where the study drug infusion was terminated before the end of surgery due to bradycardia and hypertension. Figure 1 shows the flow chart of the study.

There were no significant differences in the demographic and intraoperative data between the Phenylephrine (Group A) and Control groups (Group B) (Table 1).

**Table 1:** Demographic and intraoperative data.

Variable	Phenylephrine group (n = 55)	Control group (n = 59)	*P* value
Age (yr)	34.02 ± 4.25	33.68 ± 4.32	.673
Weight (kg)	74.98 ± 12.59	71.81 ± 10.87	.152
Height (m)	1.57 ± 0.04	1.56 ± 0.03	.644
BMI (kg/m^2^)	30.29 ± 4.78	29.11 ± 3.94	.153
Intraoperative ambient temperature (°C)	20.47 ± 0.84	20.33 ± 0.86	.406
Recovery area ambient temperature (°C)	22.19 ± 1.09	21.98 ± 0.95	.279
Time between spinal block and starting drug infusion (min)	1 [1–3]	2 [1–3]	.433
Time between starting drug infusion and skin incision (min)	4 [3–8]	5 [3–8]	.628
Duration of surgery (min)	46 [37–57]	45 [38–57]	.840
Volume of fluids administered (mL)	800 [500–900]	800 [550–1000]	.315
Estimated blood loss (mL)	300 [200–400]	300 [200–400]	.562

Values are expressed in mean ± SD or median [IQR] where appropriate. *P* value > .05 comparing both groups.

BMI = body mass index, IQR = interquartile range, SD = standard deviation.

**Figure 2.**
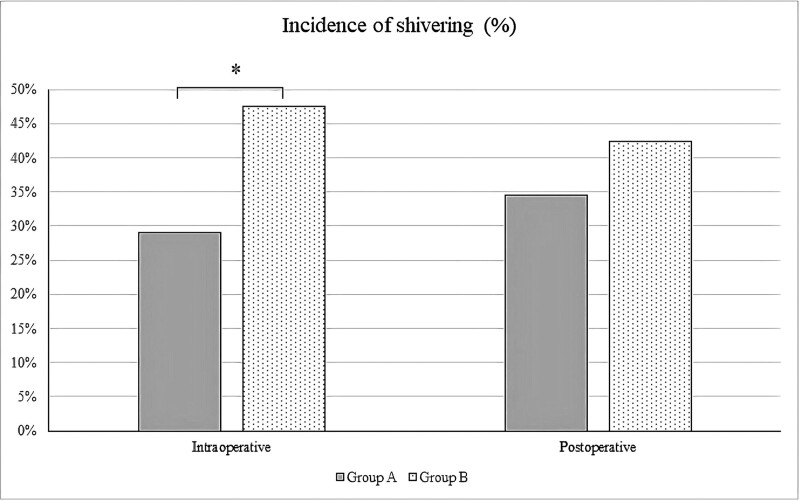
Incidence of perioperative shivering. *P value < .05 is statistically significant. (Group A: Phenylephrine Group, Group B: Control Group).

The incidence of perioperative shivering is shown in Figure 2. Intraoperatively, the incidence of shivering was lower in the Phenylephrine Group (29.1%) than in the Control Group (47.5%; *P* = .044). Postoperatively, the incidence of shivering was lower in the Phenylephrine group (34.5%) than in the Control Group (42.4%); however, this difference was not statistically significant (*P* = .391).

Table 2 shows the intensity of shivering. The intensity of shivering was comparable between the groups perioperatively.

**Table 2:** Perioperative shivering score.

Time	BSAS score	Phenylephrine group, n (%)	Control group, n (%)	*P* value
After spinal block	0	55 (100%)	53 (89.8%)	.052
0 min	1	0	3 (5.1%)	
	2	0	3 (5.1%)	
	3	0	0	
15 min	0	47 (85.5%)	50 (84.7%)	.927
	1	6 (10.9%)	6 (10.2%)	
	2	2 (3.6%)	3 (5.1%)	
	3	0	0	
30 min	0	40 (75.5%)	35 (59.3%)	.054
	1	4 (7.5%)	14 (23.7%)	
	2	9 (17.0%)	8 (13.6%)	
	3	0	2 (3.4%)	
45 min	0	33 (80.5%)	28 (62.2%)	.086
	1	3 (7.3%)	11 (24.4%)	
	2	5 (12.2%)	6 (13.3%)	
	3	0	0	
60 min	0	14 (73.7%)	14 (77.8%)	.250
	1	1 (5.3%)	3 (16.7%)	
	2	4 (21.1%)	1 (5.6%)	
	3	0	0	
Recovery room	0	36 (65.5%)	35 (59.3%)	.251
0 min	1	10 (18.2%)	18 (30.5%)	
	2	9 (16.4%)	6 (10.2%)	
	3	0	0	
15 min	0	42 (76.4%)	42 (71.2%)	.821
	1	10 (18.2%)	13 (22.0%)	
	2	3 (5.5%)	4 (6.8%)	
	3	0	0	
30 min	0	43 (78.2%)	47 (79.7%)	.865
	1	10 (18.2%)	9 (15.3%)	
	2	2 (3.6%)	3 (5.1%)	
	3	0	0	

Values are expressed in number (%). *P* value > 0.05 comparing both groups.

BSAS = Bedside Shivering Assessment Scale.

**Figure 3.**
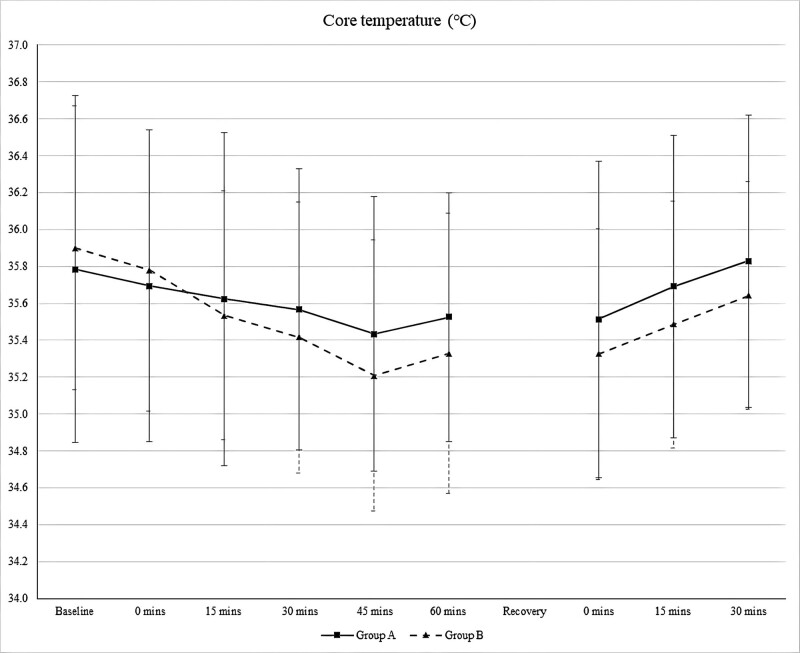
Perioperative core temperature trends. *P* value > .05 comparing both groups. (Group A: Phenylephrine Group, Group B: Control Group).

Figure 3 shows the perioperative core temperature trends in the phenylephrine and control groups. There were no significant differences in the core temperature between the groups perioperatively. Initially, a decrease in the core temperature was observed in both groups. At the end of the surgery and in the recovery room, the Control Group showed a lower core temperature than the Phenylephrine Group; however, the difference was not statistically significant. The core temperature change in the Phenylephrine Group was less steep than that in the Control Group.

**Figure 4.**
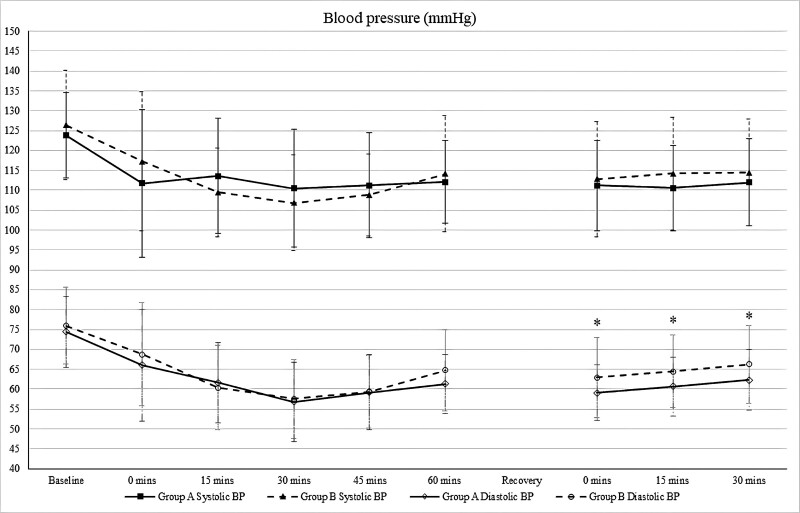
Perioperative blood pressure trends. *P value < .05 is statistically significant. (Group A: Phenylephrine Group, Group B: Control Group).

Figure 4 shows the perioperative blood pressure trends between the groups. There was no significant difference in preoperative systolic blood pressure between the groups. The phenylephrine Group showed higher systolic blood pressure from 15 to 45 minutes intraoperatively. As for diastolic blood pressure, there were significant differences between both groups postoperatively, with p values of 0.024, 0.050, and 0.026 at 0 minutes, 15 minutes, and 30 minutes, respectively.

**Figure 5.**
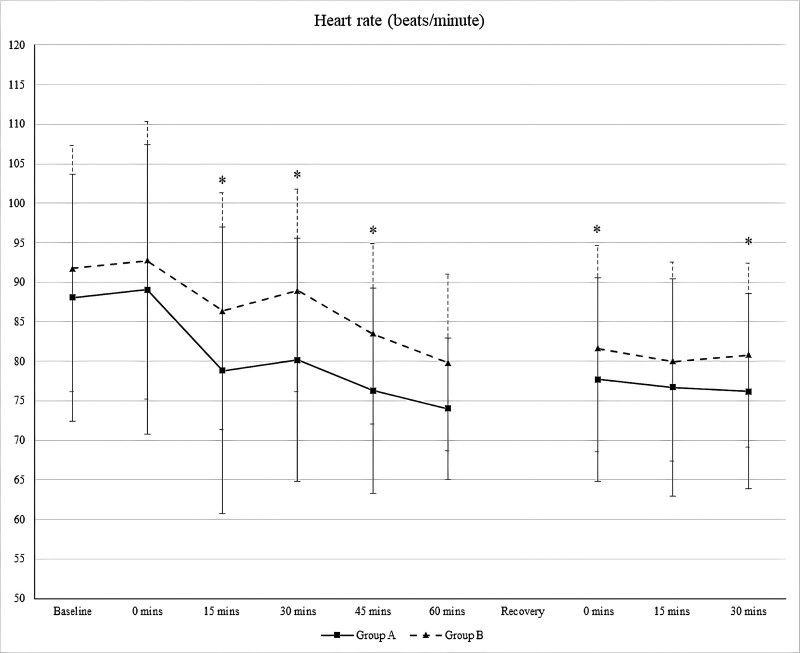
Perioperative heart rate trends. *P value < .05 is statistically significant. (Group A: Phenylephrine Group, Group B: Control Group).

Figure 5 shows the perioperative heart rate trends between the phenylephrine and control groups. The phenylephrine Group showed a lower heart rate trend throughout the study, and this was statistically significant at 15, 30, and 45 minutes after spinal block (*P* = .005, .000, and .008, respectively), and at 0 and 30 minutes (*P* value .004, .020 respectively) in the recovery room.

Five patients in the Control Group (8.5%) required rescue pethidine for shivering compared to 1 patient in the Phenylephrine Group (1.8%). However, this difference was not statistically significant (*P* = .145).

Regarding adverse events, 24 patients in the Phenylephrine Group (43.6%) and 21 patients in the Control Group (35.6%) experienced intraoperative hypotension, which was treated with ephedrine. One patient in the Phenylephrine Group (1.8%) had intraoperative hypertension. Postoperatively, 1 patient in the Control Group (1.7%) had hypertension. There were no significant differences between the groups for any adverse event (*P* = .380 for intraoperative hypotension, .298 for intraoperative hypertension, and .332 for postoperative hypertension).

## 4. Discussion

The findings from our study suggest that phenylephrine infusion significantly reduced the incidence of intraoperative shivering. The incidence of intraoperative shivering in the phenylephrine group was 29.1% compared to 47.5% in the control group. This translated to an 18.4% reduction in intraoperative shivering among the patients who received phenylephrine. A similar finding was reported by Palanisamy et al^[16]^, who reported an incidence of shivering of 24% in the phenylephrine group compared to 53% in the controlled group. Shivering primarily occurs in response to core hypothermia. When the core temperature decreases to a certain point, known as the shivering threshold, thermoregulatory shivering occurs.^[1]^ A reduced incidence of shivering in the phenylephrine group was observed up to the recovery area, although the data were not statistically significant.

The mechanism by which phenylephrine reduces shivering remains unknown. Shivering is commonly associated with hypothermia. Central neuraxial anesthesia reduces the vasoconstriction and shivering thresholds by approximately 0.6℃ when measured above the level of block.^[1]^ During the first hour of neuraxial anesthesia, the core temperature decreased by 0.8 ± 0.3 °C, which was mainly due to heat redistribution from the core to the peripheries due to vasodilation from neuraxial anesthesia.^[17]^ Phenylephrine is an α1 agonist that causes vasoconstriction and may limit the core to peripheral redistribution of body temperature following spinal anesthesia and reduce hypothermia and shivering.^[13]^

Our study did not find any significant difference in core temperature between phenylephrine and control group. Even though the decrease in mean core temperature from baseline at the end of surgery was lower in the phenylephrine group (0.26℃) than in the control group (0.57℃), this data was not statistically significant. This finding was contrasted to 3 of other studies.^[13,15,16]^ Ro et al^[13]^ studied patients undergoing orthopedic surgery under spinal anesthesia and found that phenylephrine infusion reduced the magnitude of core hypothermia. Hilton et al^[15]^ and Palanisamy et al^[16]^ found that phenylephrine attenuated maternal hypothermia in patients undergoing cesarean section under spinal anesthesia. Both studies postulated that the thermoprotective effect of phenylephrine was due to α1-mediated vasoconstriction, which was demonstrated in a study that found the presence of vasoconstrictor-mediated thermogenesis in skeletal muscles in vitro.^[18]^

Phenylephrine increases systemic blood pressure by increasing the systemic vascular resistance.^[13]^ This was in accordance with our findings, in which patients who received phenylephrine demonstrated a higher systolic blood pressure 15 minutes after surgery. This effect of phenylephrine is beneficial for treating hypotension, and in clinical practice, phenylephrine has been widely used to treat hypotension in cesarean section.^[12,19,20]^ Hypotension during spinal anesthesia could be due to the vasodilatory effects of the spinal block, which reduces systemic vascular resistance.^[21]^ Spinal block also causes a decrease in venous vasomotor tone, which increases venous pooling and consequently reduces venous return, thereby decreasing cardiac output.^[22]^

Phenylephrine causes bradycardia in response to a physiological baroreceptor reflex.^[23]^ Patients who received phenylephrine demonstrated a lower heart rate trend throughout surgery, and this effect persisted in the recovery room even after stopping the infusion. Although the difference in heart rate between both groups was statistically significant at 15 to 45 minutes postspinal block, and at 0 minutes and 30 minutes in the recovery room, the heart rate trend in the phenylephrine group was above 70 beats per minute, which suggested that it is safe to use phenylephrine infusion in cesarean section. Previous studies have recommended titrating phenylephrine infusion to maintain blood pressure near baseline levels to better tolerate a relatively lower heart rate.^[12,19]^

This study had a few limitations. First, this study was conducted only among the maternal population who underwent lower segment cesarean section; hence, it is uncertain whether the type of surgery had any influence on the results. The presence of other factors, such as anxiety, could contribute to shivering, which we did not consider in our study. In addition, the primary outcome (shivering) was measured at 15-minute intervals in our study, but changes might have occurred between measurements, which may have influenced the trend of the results. Finally, we did not determine the optimal phenylephrine dose required to effectively reduce perioperative shivering. We recommend that phenylephrine be studied in different population groups with regard to perioperative shivering. Further studies are required to determine the optimal dose of phenylephrine to prevent shivering.

## 5. Conclusion

We conclude that phenylephrine infusion reduces the incidence of perioperative shivering in lower segment cesarean sections under spinal anesthesia.

## Author contributions

**Conceptualization:** Syarifah Noor Nazihah Sayed Masri.

**Data curation:** Jessica Tan Sook Kuan.

**Formal analysis:** Qurratu Aini Musthafa.

**Funding acquisition:** Jessica Tan Sook Kuan, Farah Hanim Abdullah.

**Investigation:** Jessica Tan Sook Kuan.

**Methodology:** Jessica Tan Sook Kuan, Qurratu Aini Musthafa.

**Project administration:** Jessica Tan Sook Kuan.

**Software:** Qurratu Aini Musthafa.

**Supervision:** Syarifah Noor Nazihah Sayed Masri.

**Validation:** Qurratu Aini Musthafa, Syarifah Noor Nazihah Sayed Masri.

**Visualization:** Farah Hanim Abdullah, Syarifah Noor Nazihah Sayed Masri.

**Writing – original draft:** Jessica Tan Sook Kuan.

**Writing – review & editing:** Farah Hanim Abdullah, Syarifah Noor Nazihah Sayed Masri.
